# Bamboos for weaving and relevant traditional knowledge in Sansui, Southwest China

**DOI:** 10.1186/s13002-020-00418-9

**Published:** 2020-10-20

**Authors:** Binsheng Luo, Selena Ahmed, Chunlin Long

**Affiliations:** 1 College of Life and Environmental Sciences, Minzu University of China, Beijing, 100081 China; 2 Key Laboratory of Ethnomedicine (Minzu University of China), Ministry of Education, Beijing, 100081 China; 3 The Food and Health Lab, Department of Health and Human Development, Montana State University, Bozeman, MT 59717 USA; 4 Kunming Institute of Botany, Chinese Academy of Sciences, Kunming, 650201 China

**Keywords:** Bamboo diversity, Bamboo weaving, Traditional handicraft, Ethnobotany, Miao ethnic group, Governance

## Abstract

**Background:**

Traditional bamboo weaving has been practiced for centuries in Sansui, a county dominated by the Miao people, in Guizhou province of Southwest China. Sansui bamboo weaving represents an intangible cultural heritage as defined by UNESCO, but, like many other traditional handicrafts in China, it has suffered a downfall in this period of rapid development. Sansui bamboo weaving is now experiencing a renaissance due to the joint efforts of the local government, bamboo weaving companies, and individual bamboo weavers. However, what bamboo species have supported the traditional bamboo weaving in Sansui keeps unknown up to now. The traditional knowledge and technology associated with bamboo weaving have not been reported. In addition, the resumption of the local bamboo industry may provide some valuable experiences for other downfallen traditional handicrafts or local communities. Thus, an ethnobotanical study on Sansui bamboo weaving has been carried out.

**Methods:**

This study mainly used ethnobotanical methods, including key informant interviews and participatory observations. Different stakeholders were selected by applying the snowball method as our key informants including 6 officials, 37 bamboo weavers, and 17 bamboo and bamboo weaving product merchants. We also went into the local weavers’ houses to visit the whole weaving process. The bamboo and dye plant species for bamboo weaving were identified by taxonomists and referring to online databases available.

**Results:**

Based on field investigations, 17 bamboo species used for weaving were recorded. Different bamboo species were woven for different purposes based on their own characters. *Phyllostachys heteroclada* is the most popular species locally. Bamboo strips are usually dyed by using *Platycarya strobilacea* and *Rubia cordifolia* to be made for different images. In recent years, the size, functions, and materials of local bamboo weaving crafts as well as their market mode have been changed to adapt to new development trends and to cater to the market. In addition, the cooperation among bamboo weavers, bamboo companies, and household workshops has provided great support to the local bamboo industry and to reboot the economy of the local community. Some suggestions for the sustainable economic development of Sansui bamboo weaving and other Chinese traditional handicrafts are proposed.

**Conclusion:**

In the present study, the bamboo weaving-associated traditional knowledge was collected by means of ethnobotanical methods. The recent renaissance of the bamboo weaving business in Sansui can be attributed both to government support and the innovations of the bamboo weaving industry itself. The developing mode (“Internet + intangible cultural heritage + poverty alleviation”), which combined the internet, poverty alleviation, and intangible cultural heritage, is valid and worth being promoted.

## Background

Sansui is a county in the southeast of Guizhou province, China. It is famous for its local bamboo weaving products, which have a 400-year history and are nationally renowned for their exquisite shape, wide varieties, and practical utility. The Chinese government has paid great attention to Sansui bamboo weaving since the establishment of the country. In 1984, the leader of the People’s Republic of China, Deng Xiaoping, presented a Sansui bamboo hat to the US president Ronald Reagan during his official visit to China. Moreover, Sansui bamboo weaving was formally recognized by the government of Guizhou province as an “intangible cultural heritage” in 2007 [1]. In 2008, Sansui County was named as one of the “Chinese Folk Culture Art Villages” by the Ministry of Culture of China [1].

For people who live close to the bamboo forests in Sansui, bamboo weaving is already integrated into their daily lives. Almost every local person, from young children to octogenarians, is skillful at cutting bamboo sticks into splits and at weaving. The bamboo splits can be woven into various items, from everyday objects to children’s toys. However, according to the local informants, after China carried out reform and open policy (1980s), the commercialization of production and the modernization of people’s daily lives have made a considerable progress. Accordingly, traditional bamboo weaving in Sansui has faced a series of problems similar to those faced by other traditional handicrafts or skills. Many bamboo products have been replaced by products made with other materials, particularly plastics, which are often cheaper or have desirable properties. Therefore, the demand for traditional bamboo wares is not as high as it was in the past. Some have even suggested that the traditional bamboo weaving industry might not fit current economic development trends with its hallmarks of digitization, standardization, and the speed of production [2].

To the best of our knowledge, only one research paper has analyzed the current state of Sansui bamboo weaving and offered suggestions for strengthening this local industry [1]. Some other papers only discussed bamboo resources in Guizhou province but not bamboo weaving per se [3, 4], while others focused on the bamboo industry in Guizhou province from the perspective of industrial ecology [5–7]. Sansui bamboo weaving had its history of glory decades ago; like many other traditional handicrafts, it suffered a decline from the modernization. However, it has been keeping up with the times nowadays, which also caught our attention and arouse our curiosity. How local bamboo weaving has survived, adapted, and evolved despite the rapid modernization in the last three decades is the focus of this study. In addition, bamboo species that supported the traditional bamboo weaving in Sansui keeps unknown up to now. The traditional knowledge about the plant basis of Sansui bamboo weaving had not been reported either.

Thus, in order to more fully understand the considerable changes to traditional bamboo weaving in Sansui and its management pattern, as well as to document related ethnobotanical knowledge, an integrated investigation was carried out in Sansui County in 2016 and 2017. This study can, therefore, provide broadly applicable insights for protecting and developing traditional handicrafts in the context of economic changes. The ethnobotanical investigation can also help to record and protect the bamboo weaving knowledge from disappearing.

## Methods

### Study area

**Fig. 1:**
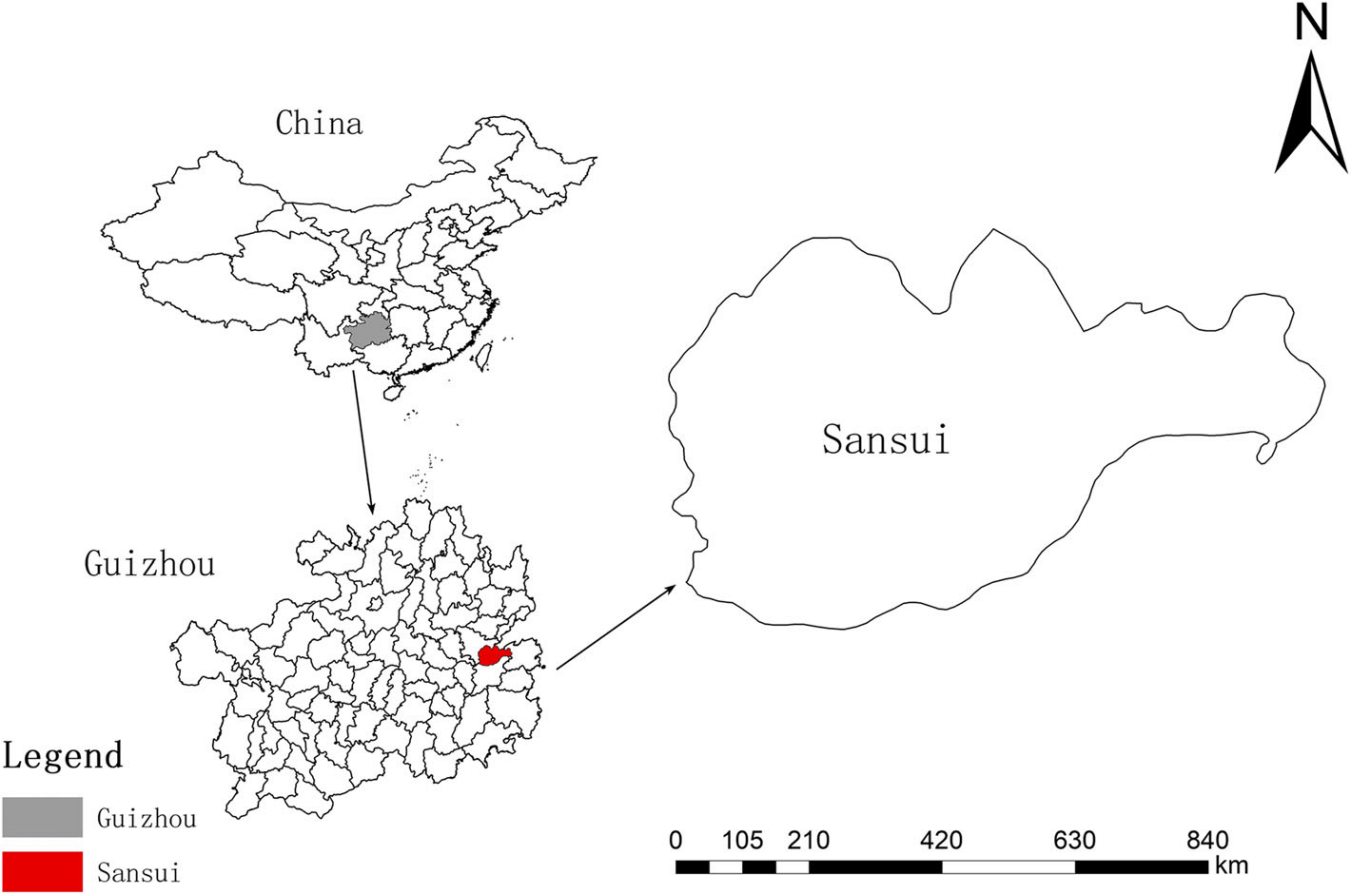
The study area: Sansui County in Guizhou province, China

Sansui County is located in the Qiandongnan Miao and Dong Autonomous Prefecture in Guizhou province of Southwest China (Fig. 1). More than 70% of the population are minority people, and the majority of them belong to the Miao and Dong ethnic groups [8]. The area is characterized by a warm climate, plentiful rainfall, abundant sunshine, and rainy seasons, which coincide with high temperatures [9]. Due to this climate, together with fertile soil, wild bamboo grows very well in Sansui County. Thus, the wild bamboo resources are quite abundant in Sansui, providing excellent raw material for bamboo weaving products [10].

### Data collection

Several field investigations were conducted in 2016 and 2018 mainly using ethnobotanical methods, including key informant interviews and participatory observations [11–13]. Different stakeholders were chosen as our key informants including 6 officials, 37 bamboo weavers, and 17 bamboo and bamboo weaving product merchants. All 60 informants (30 males and 30 females) were selected by applying the snowball method and provided informed consent before the interviews [11]. The questions we asked in the interviews mainly included the following: (1) What plant (bamboo species) do you use? (2) Why do you choose this species (characters)? and (3) What changes happened to the bamboo weaving products and local bamboo weaving industry by comparing with before?

The field investigations mainly included two parts: market surveys and individual interviews. The market for trading bamboo and woven bamboo goods usually begins remarkably early at about 2:00 am and ends at about 10:00 am. The most crowded time is from about 3:00 to 5:00 am. According to our interviews, in the old times, sellers were usually from very remote areas which required a lot of time on traffic in mountainous areas like Sansui. Thus, those sellers set out at the daytime and arrived at the market at night as early as possible to compete for a better spot for selling. In addition, the transport of bamboo and bamboo products at night were less crowded. This special time schedule has been kept up until now. The market is not open on the 8th, 18th, or 28th of each month according to the Chinese lunar calendar, while the volume of trade is the greatest on the 4th, 14th, and 24th of the Chinese lunar calendar. According to some of the merchants, local people chose these days because they are easy to remember. Our market investigations were carried out before dawn and into the late morning. The types, characters, prices, and other information on bamboo and bamboo products were recorded.

In addition to conducting market interviews, we also went to different villages to interview local bamboo weavers, and we participated in weaving to learn more about the process and materials. With the assistance from the interviewees, we got access to different bamboo materials in their working places and in the wild. The bamboo and dye plant species for bamboo weaving were identified according to morphological characters and geographical origins using taxonomy and databases available. The electronic taxonomic database we used included *The Plant List* (http://www.theplantlist.org/) and *Flora of China* (http://www.eFloras.org/). Voucher specimens of bamboo species were collected and deposited in the Herbarium, College of Life and Environmental Sciences, Minzu University of China. These bamboo species, along with their characteristics and usages, are documented in Table 1.

**Table 1:** Plant species used for bamboo weaving in Sansui County

Scientific name	Bamboo split characteristics	Usage
*Bambusa distegia* (Keng & Keng f.) L.C.Chia & H.L.Fung	Flexible	Weaving bamboo mat
*Bambusa emeiensis* L.C.Chia & H.L.Fung	Flexible	Farm tools, bamboo handicrafts, horticultural plants, edible shoots and tastes bland
*Bambusa pervariabilis* McClure	Solid and straight	Building materials, farm tools, handicrafts, furniture, medicine (bamboo shavings)
*Dendrocalamus farinosus* (Keng & Keng f.) L.C. Chia & H.L.Fung	Flexible	Farm tools, bamboo handicrafts, horticultural plants, edible shoots
*Dendrocalamus latiflorus* Munro	Flexible	Building architectures, handicrafts, horticultural plants, edible and sweet shoots
*Fargesia semicoriacea* T.P. Yi	Slender and firm	Bamboo sieve
*Lingnania intermedia* (Hsueh f. & T.P.Yi) T.P. Yi	Flexible	The embroidered border on the different bamboo containers
*Phyllostachys bambusocides* Siebold & Zucc.	Firm and hard	Bamboo dustpan
*Phyllostachys edulis* (Carrière) J.Houz.	Flexible	Building architectures, bamboo handicrafts, paper, farm tools, horticultural plants, edible shoots
*Phyllostachys glauca* McClure	Firm and flexible	Bamboo handicrafts, farm tools, edible shoots
*Phyllostachys heteroclada* Oliv.	Flexible	Elaborate bamboo handicrafts, edible shoots
*Phyllostachys heterocycla* (Carrière) Matsum.	Big and hard	Building architectures, bamboo handicrafts, broom, paper, edible shoots
*Phyllostachys mannii* Gamble	Firm and flexible	Bamboo basket and bamboo mat, forestation species
*Phyllostachys meyeri* McClure	Firm	Umbrella slotware, bamboo handicrafts, edible shoots
*Phyllostachys nidularia* Munro	Fragile	Fence, lobster basket, horticultural plants, edible shoots
*Phyllostachys sulphurea* (Carrière) Rivière & C. Rivière	Slender and firm	Excellent material for bamboo sieve
*Pleioblastus simonii* (Carrière) Nakai	Flexible	Bamboo handicrafts, fishing rod, edible shoots but bitter

## Results and discussion

### The diversity of bamboo materials in bamboo weaving

During our research in Sansui County and the surrounding areas, plentiful bamboo forests were observed. Almost all species used for bamboo weaving could be collected in Sansui County. According to an investigation by Wu [4], the total area of bamboo forests in Qiandongnan Miao and Dong Autonomous Prefecture is around 190 km^2^. Local weavers use different bamboo materials for different purposes while weaving bamboo products according to our communication with local bamboo weavers. For example, an elaborate bamboo hat, which can prevent the wearer from raindrops, requires flexible bamboo splits, and *Phyllostachys heteroclada* is the best choice for this purpose. On the other hand, to make the best mat for drying grain requires flat and wide bamboo splits, so the bigger and more solid *Phyllostachys heterocycle* is the most suitable choice. Based on our field investigations, 17 bamboo species belonging to 7 genera in the family Poaceae were documented (Table 1), including scientific names, characteristics, and usages. These investigations demonstrated a great diversity of bamboo materials, which reflects both the rich bamboo biodiversity in the region and the complexity of Sansui bamboo weaving.

Taxonomically, over half of the species in this investigation are in the genus *Phyllostachys*. This illustrates that bamboo species from *Phyllostachys* are popular locally, which may be due to the better properties of the bamboo splits from these species. *Phyllostachys edulis* has a long planting history, the largest plantation area, and the greatest economic value in China. It is frequently used to weave different bamboo products in many other places in South China. However, the native bamboo weavers in Sansui prefer to use *Phyllostachys heteroclada* splits rather than *Phyllostachys edulis* splits for weaving. *Phyllostachys heteroclada* is the most frequently used bamboo material owing to the following natural properties: (1) most importantly, *P. heteroclada* splits are flexible and do not easily snap when weaving; (2) breaking the bamboo stick into bamboo splits is a key step prior to weaving, and it is easier for *P. heteroclada* to be made into bamboo splits than it is for other species; (3) the color of *P. heteroclada* is deemed beautiful, unlike the color of *Fargesia semicoriacea*, which looks pale; (4) *P. heteroclada* is easier to procure because it is widely available across southeast Guizhou province, including Sansui County.

The number of species used for different purposes is listed in Table 2. Making handicrafts and consuming as food make up the highest usage percentage (58.8%) with ten species. Most bamboo species used for handicrafts are required to be flexible. Also, bamboo shoots are a traditional Chinese food with thousands of years of history. Eating bamboo shoots is popular and healthy because they contain high levels of plant fiber and very few calories [14]. The shoots from different species also have variable tastes. For example, *Dendrocalamus latiflorus* shoots taste sweet, *Bambusa emeiensis* shoots taste bland, and *Pleioblastus simonii* shoots taste bitter.

**Table 2:** The species number for different usages

Usages	Species	Percentage
Handicrafts	10	58.8
Food	10	58.8
Farm tools	7	41.2
Ornamentals	5	29.4
Architectures	4	23.5
Others	7	41.2

Seven bamboo species (41.2%) were used to make farm tools, including bamboo sieves, bamboo dustpans, and lobster baskets. The number of species used for ornamental and architectural purposes was five (29.4%) and four (23.5%), respectively. Additionally, seven species were for other purposes. *Lingnania intermedia* is usually woven into an embroidered border on different bamboo containers. The bamboo shavings of *Bambusa pervariabilis*, which are called “Zhu Ru” by local people, can be used as medicine for clearing heat (i.e., the TCM disease category) and for stopping bleeding. However, in traditional Chinese medicine, “Zhu Ru” usually refers to the shavings of *Bambusa tuldoides*, *Phyllostachys nigra*, and *Sinocalamus beecheyanus* var. *pubescens*. Whether *Bambusa pervariabilis* possesses similar medicinal effectiveness to the traditional “Zhu Ru” will require further chemical and pharmacological studies.

### Dye plants for bamboo weaving

Many ethnic groups have traditions for dyeing with natural pigments made from plants, whether for esthetics or cultural implications like the wishes for good harvest, health, and wealth, or the images of beings that people worship to. Traditional dyeing is especially widespread in Southwest China (e.g., five-colored rice used by the Zhuang people and blue-dyed cloth used by the Miao people) [15]. In this study, we found that bamboo splits were dyed and woven into bamboo handicrafts as auspicious images, characters, flowers, and animals worshipped by local people. When dyeing bamboo splits, chemical dyes are easily washed off and are harmful to humans. Comparatively, natural plant dyes can be less harmful and have longer-lasting color. Typically, local people use the bark and leaves of *Platycarya strobilacea* to dye bamboo splits for a black color. They firstly put the plant materials and bamboo splits together in boiling water for 12 h, and then they bury the splits in the soil for 48 h to fix the color. They also use *Rubia cordifolia* as a red dye using almost the same dyeing process. Furthermore, in order to weave different images, local bamboo weavers can also apply splits of various bamboo species with different colors to highlight the images they want.

### The status and transition of Sansui bamboo weaving

The whole process of bamboo weaving is detailed and complicated, which means bamboo weaving is time-consuming and, from the perspective of modern productivity, inefficient. For example, based on our participatory observation, it takes at least 5 h to finish a bamboo weaving product. Additionally, according to the local weavers, normal bamboo weaving products were in low profit and low demand in the market during the past few decades. Sometimes buying good bamboo material would also increase the cost for bamboo weavers. By only weaving bamboo products for a living, it was difficult to feed one’s family. Therefore, very few young people were willing to learn the art of bamboo weaving or choose it as a career. Consequently, traditional knowledge of bamboo weaving had been fading away.

However, in the market, we found some positive changes and innovations in the appearance and intended uses of Sansui bamboo weaving products. In the past, most bamboo weaving products were produced for very predictable purposes, such as farming equipment like bamboo hats, basket carriers, winnowing fans, and creels. However, a large number of Sansui bamboo weaving products sold on the market nowadays are ornamentals, handiworks, and even children’s toys. Interestingly, many bamboo weaving products looked exactly like old-style farming tools but in much smaller sizes and for different purposes. For instance, a regular size of creel is sold for about 3 dollars, while a small-size creel is sold as a vase for about 9 dollars. A small-size winnowing fan is marketed as a bamboo plate to place fruits, and its price (about 9 dollars) is also almost the triple of a regular winnowing fan. Additionally, some smaller bamboo wares are sold as artworks purely for esthetic and ornamental purposes. Even though only the sizes of bamboo products are smaller, according to the local sellers, people seem to be willing to pay a much higher price for them.

**Fig. 2:**
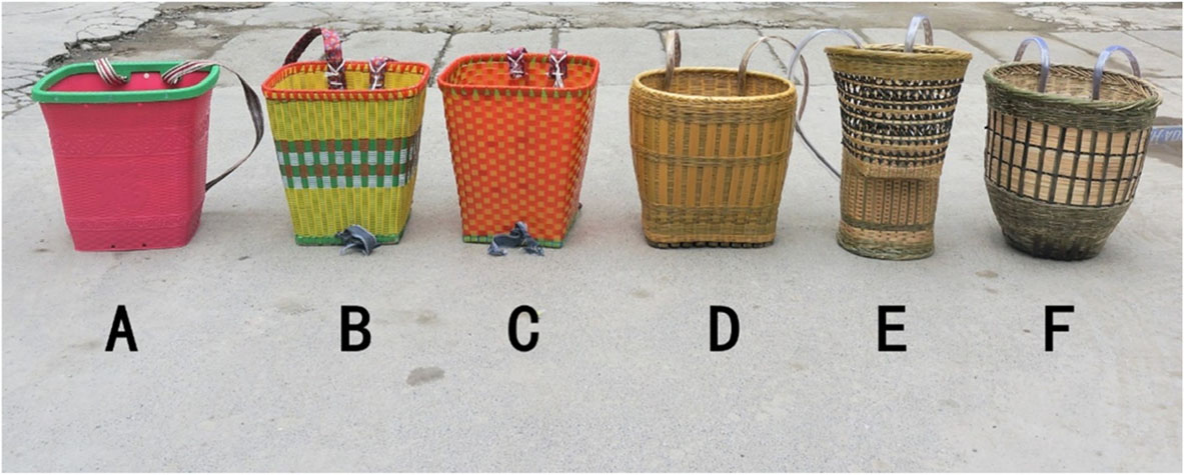
The bamboo weaving baskets on the market

**Fig. 3:**
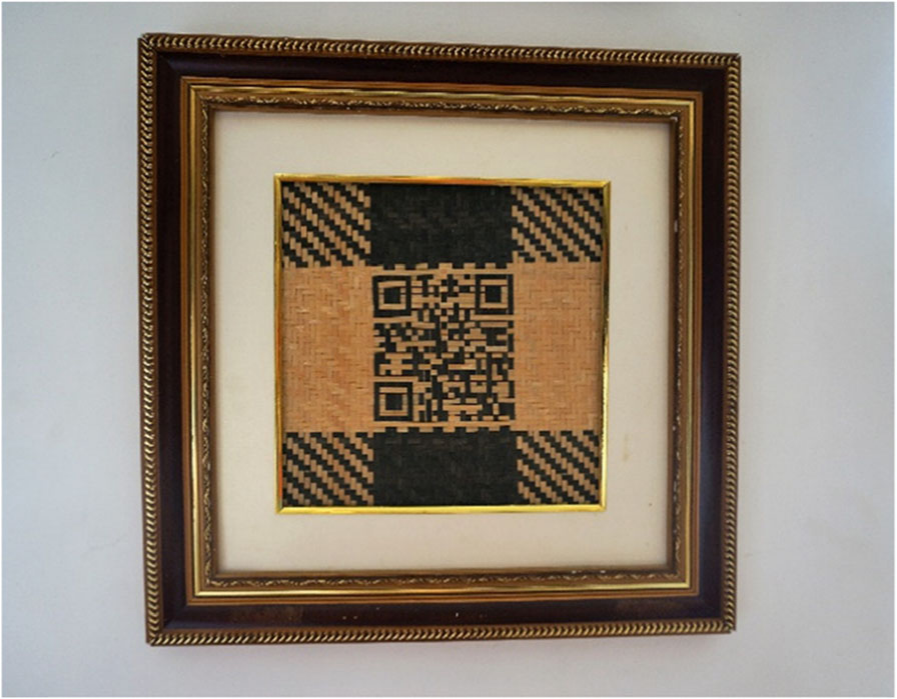
The bamboo QR code hanging on the wall

We also found that bamboo weaving is becoming fashionable by incorporating modern stylistic elements. During the market surveys, we collected different versions of pack baskets for carrying babies, ranging from the most traditional styles to the most modern ones (Fig. 2). In Fig. 2, baskets D, E, and F are traditional baskets, but their straps are covered with transparent plastic hoses to protect the inner bamboo straps and provide comfort. Baskets B and C are woven with plastic strips but using traditional weaving methods, and their straps are made of colorful cloth. Because of the plastic materials, these products are more robust but also heavier than traditional bamboo products. Most differently, basket A is totally plastic and made by using molds instead of weaving. Decorative patterns make basket A look like a real bamboo weaving product. From basket A to basket F, a combination of modern elements and traditional bamboo weaving methods can be observed. The local weavers also make a series of exquisite bamboo living supplies like handbags, lampshades, calligraphy, paintings, and others, which show the evolution and innovation of bamboo weaving. Some modern slogans and graphic patterns representing good wishes are also woven into bamboo products by the local people. We even found a bamboo-made painting hung on the wall, which was a two-dimension code (QR code) (Fig. 3). It can be scanned by smartphones to auto-access the website of the online shop.

In our market surveys of woven bamboo products, we found many changes to traditional products, including the appearance, size, intended use, and materials used. These changes and the creation of new bamboo weaving products used in modern life could solve the problem of low demand, which shows the wisdom of local bamboo weavers in responding to a changing economy. Going with the tides and innovating on traditions might be basic survival strategies for Sansui bamboo weaving, as well as for other traditional handicrafts in China in such a rapidly developing era.

### The development of Sansui bamboo weaving

According to our informants, the development of the bamboo weaving in recent two decades was mainly driven by both government and local young entrepreneurs. In the macro policy of rural poverty alleviation in China, local governments and entrepreneurs respected the potential values of the traditional bamboo weaving for creating more jobs and making more economic contributions.

Traditionally, most bamboo weaving businesses are run by individual households. In the beginning, the primary purpose of weaving bamboo products was to achieve self-sufficiency. The weakness of the domestic family workshop has been shown with the changing times and has led to the downfall of traditional Sansui bamboo weaving and other traditional handiworks in China. However, during our field research, we found that Sansui bamboo weaving has stepped into a new era, in which the local government has played an important role.

In recent decades, many local men went into urban areas to make more income, leaving women at home to look after the children and to do housework. In order to help those women, make extra money to supplement the family income, the local government has collaborated with bamboo weaving companies to teach these women bamboo weaving skills. This policy was accessible and attractive, according to our interviews. Each woman can get 60 yuan (almost 9 US dollars) every day as a subsidy from the local government for participating in the bamboo weaving training workshops. Also, the trainers from the bamboo weaving companies can get 200 yuan each day as the teaching payment. The women trained from these workshops can make the bamboo weaving products while staying at home. The bamboo products will be examined and purchased by the bamboo weaving companies if they meet the criteria. In this way, local families can earn extra money, while companies can expand the production of bamboo weaving products and make more profits. This pattern of cooperation between the government and companies follows the targeted poverty alleviation policy in China. Thus, the local government helps the bamboo weavers learn about innovative weaving methods and new products free of charge and often organizes activities for weavers to communicate with and learn from each other. The government also supports outstanding bamboo weavers to participate in associated expos and exhibitions out of the county for learning and sharing. These contributions from the local government not only help in the conservation of traditional bamboo weaving, but they also bring new vitality to this craft in the modern era.

Another change is in the sales model of bamboo weaving producers. Instead of operating as comparatively weak domestic family workshops, producers operate as companies that bring together many families and skillful weavers, thus allowing them to sell bamboo products to a nationwide market. In this way, bamboo weavers can get many more orders and keep a certain percentage of profits from their bamboo products. As for marketing channels, the companies can sell their products both online and in-person. For online marketing, companies can take full advantage of internet applications: they own stores both on Taobao, the biggest online shopping website in China, and WeChat, the most popular social network application in China. For in-person marketing, they own the stores on the local commercial pedestrian street, which can keep a steady source of customers. Compared to the traditional individual household pattern, the current company based one is more convenient and reliable. It has brought a new vitality to traditional bamboo weaving.

### The developing pattern analysis: “Internet + intangible cultural heritage + poverty alleviation”

China is a developing country with about 70 million poverty-stricken individuals in 2017, which is still falling behind developed countries [16]. Sansui County has been identified as a national-level poverty-stricken county since 2012 by the Steering Committee Office of Poverty Alleviation and Development of State Council of China [listed on its official website http://www.cpad.gov.cn/art/2012/3/19/art_50_23706.html]. In recent years, the local government has made highly effective progress: the poverty-stricken population in Sansui County has been reduced from about 90,000 individuals in 2011 to 25,000 individuals in 2017. Correspondingly, the poverty incidence has declined from 46.3% in 2011 to 12.8% in 2017, according to the Sansui government work report listed on their official website on August 3, 2018 (http://www.gzss.gov.cn/xwzx/tzgg/201708/t20170804_2763970.html).

For the past few years, transportation around Sansui County has been improved significantly by the construction of high-speed railways and expressways, which have created a suitable environment for the development of the local express delivery industry and electronic commerce. Based on these circumstances, the local government has employed a new pattern called “Internet + intangible cultural heritage + poverty alleviation” for both developing traditional bamboo weaving and targeted poverty reduction. This mode is now adopted nationwide by the Chinese central government.

In the case of bamboo weaving, e-commerce can open a new marketing channel and increase the sales for bamboo weaving products. This development pattern has also operated efficiently in targeted poverty alleviation. According to statistics obtained during interviews with local government officials, the online order volume of bamboo weaving products is more than 2500 (orders) per month, and the associated sales amount is more than 40,000 Chinese yuan (around 5700 USD). Just one bamboo weaving product company alone can help more than 40 poverty-stricken families earn 3000 Chinese yuan (around 440 USD) per family per month. Meanwhile, the problem of losing bamboo weaving skills has also been solved. For example, in Wazhai, a town in Sansui County, with the support of local government and with leadership from local senior bamboo weaving artists, the amount of bamboo weaving workshops has increased to 280. The yearly output of bamboo weaving products in Sansui County is more than 4,000,000, which is worth about 10,000,000 Chinese yuan (around 1,440,000 USD).

Traditional handicrafts usually possess rich cultural properties and extensive marketing prospects [2]. Based on the case in our study, we envision that the development of traditional handicrafts can be combined well with poverty alleviation in the context of a government targeted poverty alleviation program. Targeted poverty alleviation is not just an economic subsidy, but rather, it is a fundamental solution to the livelihood problems of poor households. This typical example echoes an old Chinese saying: “Give a man a fish, and you feed him for a day. Teach him how to fish, and you feed him for a lifetime.” As a matter of fact, similar examples have also made great success like the traditional handmade herbal incense in Tibet Region [17]. All of these cases proved that this developing pattern of “Internet + intangible cultural heritage + poverty alleviation” also can be taken as an excellent model for other places in China in the future.

### Suggestions and future perspectives

Most of the traditional handicrafts in China are facing similar challenges to the ones face by Sansui bamboo weaving in this fast-changing society [18]. Based on our investigations and literature review, we provide some suggestions for both Sansui bamboo weaving and other traditional handicrafts in China.

Importantly, maintaining the sustainability of raw materials is the fundamental first step for ensuring a sustained supply of materials. For Sansui bamboo weaving, growing bamboo can bring extensive ecological benefits, economic value, ornamental value, and even cultural value [19, 20]. Ecologically, bamboo plantations can enhance land coverage and ecosystem services [21]. Unlike other Poaceae plants, bamboo species are woody plants, allowing bamboo to become useful for timber. Bamboo plantations neither adversely effect other plants nor degrade vegetation if they were in a proper manual management [22]. As for the economic value, bamboo can be used as food or medicine and for products like handicrafts, paper, furniture, and culture [23, 24]. Since ancient times, bamboo has deeply affected the Chinese people because of its elegant shape and the excellent characters it stands for. It possesses, therefore, a great deal of cultural and ornamental value [25]. Currently, the Sansui government is building ecological bamboo gardens as a part of urban ecological construction to not only beautify the county but also to enrich the local people’s lives. As a new trend, the development of the bamboo growing and processing industry by local government could be very beneficial for local livelihood and environment, as well as for maintaining bamboo weaving traditions.

The case of Sansui bamboo weaving emphasized the importance of innovation, which can attract buyers. This model is also a pivotal solution to create more income to maintain the survival and the development of traditional handicrafts. In order to inspire more innovation, using the power of youth in universities and traditional native artists as intellectual resources could be an effective solution. Moreover, increasing publicity is also useful for attracting more potential customers. In this case, the Sansui government can promote the provenance of bamboo weaving and take full advantage of the internet and public media to expand the influence of local bamboo weaving. Additionally, the traditional handicraft industry can also obtain more consumers by collaborating with the local tourist industry.

## Conclusion

In our ethnobotanical and participatory surveys, we recorded a total of 17 species belonging to 7 genera of bamboo used for bamboo weaving. Different bamboo materials are used to weave different bamboo products. *Phyllostachys heteroclada* is the most frequently used species due to its excellent properties. The local people also use *Platycarya strobilacea* and *Rubia cordifolia* to dye bamboo splits black and red, respectively.

Bamboo weaving in Sansui has gone from decline to rebirth, due to the great support by the local government and a new development mode. The mode of combining households, companies, and the government is an effective method for poverty alleviation and for contributing to local communities. Furthermore, the development of local bamboo resources can not only ensure a supply of materials for bamboo weaving, but also it can supply other products, increase income, and promote local ecological sustainability. Some suggestions for the renaissance of the traditional handicraft industry are proposed. Innovation is the key solution to bring attention and economic benefits. We believe that traditional handicraft industries, including Sansui bamboo weaving, can make more contributions to the local community in this fast-changing modern society.

## Data Availability

All data generated or analyzed during this study are included in this published article (and its supplementary information files).
